# Phase I Study of the Mutant *IDH1* Inhibitor Ivosidenib: Long-term Safety and Clinical Activity in Patients with Conventional Chondrosarcoma

**DOI:** 10.1158/1078-0432.CCR-24-4128

**Published:** 2025-03-18

**Authors:** William D. Tap, Gregory M. Cote, Howard Burris, Lia Gore, Anthony Elias, Murali Beeram, Anthony P. Conley, Diego A. Gianolio, Zhe Qu, Susan Pandya, Jonathan C. Trent

**Affiliations:** 1 Memorial Sloan Kettering Cancer Center, New York, New York.; 2 Mass General Cancer Center, Boston, Massachusetts.; 3 Sarah Cannon Research Institute, Nashville, Tennessee.; 4 University of Colorado School of Medicine and University of Colorado Cancer Center, Aurora, Colorado.; 5 START Center for Cancer Care, San Antonio, Texas.; 6 MD Anderson Cancer Center, Houston, Texas.; 7 Servier Bio-Innovation, Boston, Massachusetts.; 8 Sylvester Comprehensive Cancer Center, Miami, Florida.

## Abstract

**Purpose:**

A phase I study demonstrated that ivosidenib, a mutant isocitrate dehydrogenase 1 (mIDH1) inhibitor, showed manageable toxicity and durable disease control in patients with m*IDH1* conventional chondrosarcoma (CS). In this study, we present long-term follow-up data on the safety and clinical activity of ivosidenib in patients with m*IDH1* conventional CS from this phase I study.

**Patients and Methods:**

This phase I, open-label, dose-escalation, and expansion study assessed ivosidenib monotherapy in patients with advanced m*IDH1* solid tumors, including CS. An ivosidenib dose of 500 mg/day was identified in the dose-escalation phase and used for the expansion phase. The primary outcome was safety and tolerability. Secondary outcomes included objective response rate and progression-free survival. The database lock date for this analysis was March 18, 2024.

**Results:**

Of 168 patients with advanced m*IDH1* solid tumors receiving ivosidenib in this study, 21 patients had CS, of which 13 had conventional histology. Six (46.2%), 4 (30.8%), and 3 (23.1%) patients with conventional CS continued ivosidenib treatment for >1 year, >6 years, and >7 years, respectively. Of the 21 patients with CS, 71.4% and 28.6% had treatment-related and serious adverse events, respectively, but no serious adverse events were considered related to ivosidenib. The objective response rate for patients with conventional CS was 23.1%, and the median duration of response was 53.5 months. The median progression-free survival of patients with conventional CS treated with ivosidenib was 7.4 months.

**Conclusions:**

Ivosidenib demonstrated long-term disease control and manageable toxicity for some patients with m*IDH1* conventional CS and is under further investigation (NCT06127407).

**Translational Relevance**

Chondrosarcomas (CSs) are a group of bone cancers with poor outcomes, and the development of effective pharmacologic therapies for the treatment of CSs represents an unmet medical need. Mutations in isocitrate dehydrogenase 1 (*IDH1*) have been shown to be present in some CSs; therefore, targeting of mutant *IDH1* (m*IDH1*) using ivosidenib may be a potential option for treatment. In this phase I dose-escalation and expansion study, ivosidenib demonstrated clinical activity and was well tolerated in patients with m*IDH1* conventional CS. Ivosidenib has the potential to improve outcomes in patients with m*IDH1* advanced conventional CS who lack available treatment options.

## Introduction

Chondrosarcomas (CSs) are a group of rare primary bone cancers that account for approximately 20% of primary bone malignancies and arise from cartilage-producing cells (1, 2). CSs are referred to as conventional when the microscopic appearance bears a close resemblance to nonneoplastic cartilage, as opposed to the rare mesenchymal, clear-cell, and differentiated CSs that have specific histologic and clinical features (3). The 2020 World Health Organization Classification of Tumors of Soft Tissue and Bone categorizes CSs into eight subtypes: (i) central atypical cartilaginous tumor/conventional CS grade 1; (ii) secondary peripheral atypical cartilaginous tumor/conventional CS grade 1; (iii) central conventional CSs, grades 2 and 3; (iv) secondary peripheral conventional CSs, grades 2 and 3; (v) conventional periosteal; (vi) clear-cell; (vii) mesenchymal; and (viii) dedifferentiated CSs (4).

Approximately 85% of CSs are classified as conventional CSs (2). The observed 5-year average survival rates for patients with conventional CS has been reported to be 68.4%, but this is highly dependent on clinical features such as the grade and tumor location (5).

The typical treatment approach for curative intent of conventional CS is surgical resection, which may result in increased morbidity for patients (e.g., limb amputation, limitations in mobility, and acute/chronic pain; ref. 6). Some patients, depending on the anatomic location (e.g., the base of the skull) and clinical features of their disease, may be treated with surgery and/or radiation. Local recurrence after surgery occurs in approximately 25% of patients with CS (7), and in such cases, additional surgery should be considered (1). If the disease is unresectable at diagnosis, or after recurrence after surgery, palliative or definitive radiotherapy may be considered (1).

Conventional CSs are resistant to both chemotherapy and radiotherapy, and despite advances in the understanding of the molecular pathogenesis of this disease, there are no approved or molecularly targeted therapies specific for conventional CS (1, 2, 8). A few systemic agents have limited evidence of activity in selected bone sarcoma types; however, these agents are not approved by the health authorities and have consequently not entered standard clinical practice. Therefore, CS remains a very serious disease with limited treatment options, and the development of effective pharmacologic therapies represents an unmet medical need.

Mutations in isocitrate dehydrogenase 1 (*IDH1*) have been detected in several malignancies (9–11), and approximately 40% of conventional CSs have mutations in *IDH1* (12). IDH catalyzes the oxidative decarboxylation of isocitrate, producing ɑ-ketoglutarate. Gain-of-function mutations in *IDH1* (and *IDH2*) lead to the production and accumulation of the oncometabolite 2-hydroxyglutarate (2-HG) instead of ɑ-ketoglutarate, which causes aberrant methylation of DNA and histones (13, 14). The impact of *IDH* mutations on pathogenesis, propagation, and prognosis in CS remains heavily debated. Some studies have shown that patients with mutant *IDH* (m*IDH*) CS have worse overall survival (OS) than patients with wild-type *IDH* CS (12, 15). However, another study indicated that *IDH* mutations are associated with longer relapse-free and metastasis-free survival in high-grade CSs (16).

Targeting m*IDH1* provides a potential treatment strategy for conventional CS (17). Ivosidenib is a potent oral targeted inhibitor of mIDH1, approved as a 500 mg daily dose in the United States and European Union in m*IDH1* acute myeloid leukemia and m*IDH1* cholangiocarcinoma (18). Ivosidenib inhibits mIDH1 only and has no activity against wild-type or mutant IDH2 (19). A phase I study (NCT02073994) investigated the use of ivosidenib monotherapy in patients with advanced m*IDH1* solid tumors, including 21 patients with m*IDH1* advanced CS, 13 of which had the conventional subtype (20). Ivosidenib demonstrated manageable toxicity (without being dose limiting) and suppression of plasma 2-HG by 56.7% (range: 14.0%–94.2%) at ivosidenib dose levels ≥300 mg/day at the data cutoff of January 16, 2019 (20). Ivosidenib showed preliminary activity with a 6-month progression-free survival (PFS) rate of 39.5% in the overall study population (20). Patients within the conventional CS subpopulation benefited more than patients with dedifferentiated CS in this study, with 6-month PFS rates of 53.8% and 0.0%, respectively (20). Here, we report the long-term follow-up data collected from the phase I study, including safety, tolerability, and clinical activity of ivosidenib in patients with m*IDH1* conventional CS.

## Patients and Methods

### Study design

The design of this study has been described previously (20). Briefly, this was a phase I, multicenter, open-label, dose-escalation, and expansion study of ivosidenib monotherapy in patients with advanced m*IDH1* solid tumors, including CS, cholangiocarcinoma, and glioma. For dose escalation, ivosidenib was administered orally in continuous 28-day cycles at 100 mg twice daily and 300, 400, 500, 600, 800, 900, and 1,200 mg once daily using a standard 3 + 3 design. Maximal plasma 2-HG suppression was achieved at the 500 mg dose, which was therefore chosen for patients included in the expansion phase of this study. Patients continued treatment with ivosidenib until disease progression or development of other unacceptable toxicity, confirmed pregnancy, death, withdrawal of consent, loss to follow-up, or ending of the study, whichever occurred first. Details on dosing, pharmacokinetics, and pharmacodynamics of ivosidenib have been previously published (20, 21).

This study was conducted in accordance with the principle of the Declaration of Helsinki and the International Conference on Harmonization for Good Clinical Practice. Before the start of the study, the protocol, amendments, and informed consent were approved by the institutional review board or independent ethics committee of each study site. Written informed consent was obtained from each participant or a legally authorized representative.

### Patients

Inclusion criteria for the dose-escalation phase of this study included patients with m*IDH1* advanced CS with measurable disease by RECIST (version 1.1) that had recurred or progressed during standard therapy or had not responded to standard therapy (or for which the investigator felt there was no standard therapy; refs. 20, 22). As previously described, patients were enrolled based on local *IDH1* testing (any method), and results were centrally confirmed using next-generation sequencing (20). In the expansion phase of this study, patients with locally advanced or metastatic m*IDH1* CS not amenable to complete surgical excision that had an Eastern Cooperative Oncology Group performance status of 0 to 1 were included (20). Exclusion criteria for this study included a heart rate–corrected QT interval of ≥450 milliseconds, the use of medications known to prolong the QT interval, or other factors that would increase the risk of QT prolongation or arrhythmic events (20).

### Study assessments

The primary outcome of this analysis was safety and tolerability of ivosidenib. The secondary outcomes of this analysis included clinical activity assessed by the objective response rate [ORR; defined as the rate of complete response + partial response (PR)], rate of stable disease, and PFS. Adverse events (AEs) were assessed and reported per the Common Terminology Criteria for AEs (version 4.03) at each visit until 28 days following the last administration of study treatment, or until study discontinuation/termination, or initiation of subsequent therapy. Clinical activity was assessed using serial radiographic evaluations to evaluate response to treatment, based on RECIST (version 1.1; ref. 22).

In the expansion phase, after all patients had completed at least 22 cycles of study treatment, a protocol amendment reduced visits to every other cycle (i.e., every 56 ± 4 days), to lessen the burden on remaining patients in the study. Radiographic evaluations (CT or MRI) were performed at screening, approximately every 84 days [± 4 days; frequency was initially every 56 (± 4 days) before protocol amendment] during study treatment (independent of dose delays and/or interruptions), at any time when progressive disease (PD) was suspected and at treatment discontinuation (for patients that discontinued treatment for any reason other than PD). Drug doses were recorded at each study visit.

### Statistical analysis

The database lock date for this analysis was March 18, 2024. Time-to-event endpoints were estimated using the Kaplan–Meier method. Descriptive statistics were used for other clinical parameters.

### Data availability

Data are not publicly available because of patient privacy concerns. De-identified patient- and/or study-level clinical trial data, including the clinical study report and study protocol, will be shared, in line with the Servier Data-Sharing Policy (available at https://vivli.org/ourmember/servier/).

## Results

### Patients

This trial was initiated in March 2014, and 168 patients with advanced m*IDH1* solid tumors received ivosidenib in this study. Of these patients, 21 had CS; 13 had conventional CS, 6 had dedifferentiated histology, and 2 were unknown (20). A database lock date of March 18, 2024, was used for this long-term data analysis. Baseline characteristics of the 21 patients with CS, including mutational profiles, have been reported previously (20), and baseline characteristics of the 13 patients with conventional CS are summarized in Table 1. The representativeness of this study population is shown in Supplementary Table S1. Of the eight patients with conventional CS with baseline co-mutation data, no additional co-mutations were identified in five patients. Two patients had two or more co-mutations identified in *TERT*, *CDKN2A*, *ATRX*, and *KMT2C*. One patient had one additional co-mutation in *ATR.*

**Table 1.** Baseline characteristics of patients with conventional CS receiving ivosidenib (*n* = 13).

Characteristic	*N* (%) unless otherwise stated
Age, years, median (range)	54.0 (38.0–88.0)
Gender	
Male	9 (69.2)
Female	4 (30.8)
AJCC tumor grade (at screening)	
I	2 (15.4)
II	8 (61.5)
III	1 (7.7)
Unknown	2 (15.4)
Local *IDH1* mutation type	
R132C	7 (53.8)
R132G	2 (15.4)
R132L	1 (7.7)
R132S	1 (7.7)
Other	2 (15.4)
ECOG PS at baseline	
0	7 (53.8)
1	6 (46.2)
Previous lines of systemic therapy	
0	7 (53.8)
1	4 (30.8)
≥2	2 (15.4)
Previous radiotherapy	3 (23.1)

Abbreviations: AJCC, American Joint Committee on Cancer; ECOG PS, Eastern Cooperative Oncology Group performance status.

### Treatment in patients with conventional CS

**Figure 1.**
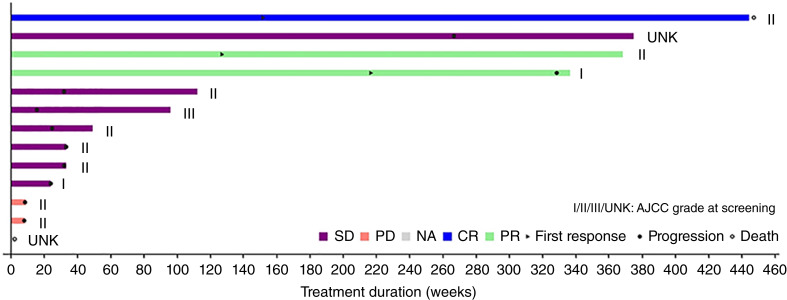
Duration of treatment with ivosidenib in patients with conventional CS. As of the database lock date for this analysis (March 18, 2024), all patients had discontinued study treatment. As per RECIST v1.1, stable disease (SD) occurring with <42 days of the first dose is assigned as unknown (UNK). The American Joint Committee on Cancer (AJCC) grade reported is at screening visit. CR, complete response; NA, not assessed.

For patients with conventional CS (*n* = 13), doses of ivosidenib received were 100 mg twice daily (*n* = 1), 400 mg once daily (*n* = 1), 500 mg once daily (*n* = 7), 800 mg once daily (*n* = 2), and 1,200 mg once daily (*n* = 2). Delays of ≥1 dose occurred in 3 (23.1%) patients with conventional CS: 1 in the 400 mg once daily group, 1 in the 500 mg once daily group, and 1 in the 800 mg once daily group. Overall, the mean duration of dose delay was 9.3 (standard deviation: 10.4) days. The median number of doses received in patients with conventional CS was 341 (range: 14–3,095), and the median relative dose intensity was 100%. Patients with conventional CS received ivosidenib for a median duration of 49.0 weeks (11.3 months; range: 2.0–444.6 weeks or 0.5–102.3 months; Fig. 1) and a mean duration of 145.4 weeks (33.4 months; standard deviation: 161.6 weeks or 37.2 months).

Six patients (46.2%) continued ivosidenib treatment for >1 year: 3 of 7 in the 500 mg once daily group, 1 of 2 in the 800 mg once daily group, and 2 of 2 in the 1,200 mg once daily group. Four patients (30.8%) continued ivosidenib therapy for >6 years, and three patients continued treatment for >7 years.

### Safety and tolerability of ivosidenib in patients with CS

All patients (21/21) experienced AEs, with 15 experiencing treatment-related AEs. The most frequently reported all-grade treatment-emergent AEs (mostly grade 1 or 2) were diarrhea (*n* = 10) and nausea (*n* = 7; Table 2). Twelve patients had grade ≥3 AEs, and one grade ≥3 AE (hypophosphatemia) was reported as possibly related to ivosidenib treatment. However, this AE resolved without treatment and did not require ivosidenib dose adjustment.

**Table 2.** Most frequently reported AEs in >15% of patients with CS receiving ivosidenib.

AE	Dedifferentiated CS (*N* = 6), *N* (%)	Conventional CS (*N* = 13), *N* (%)	Overall (*N* = 21), *N* (%)
Any AE	6 (100.0)	13 (100.0)	21 (100.0)
Treatment-related AEs	4 (66.7)	9 (69.2)	15 (71.4)
Grade ≥3 AEs	5 (83.3)	6 (46.2)	12 (57.1)
SAEs	3 (50.0)	3 (23.1)	6 (28.6)
Treatment-related SAEs	0 (0.0)	0 (0.0)	0 (0.0)
Most frequently reported AEs			
Diarrhea	2 (33.3)	6 (46.2)	10 (47.6)
Nausea	2 (33.3)	5 (38.5)	7 (33.3)
Fatigue	2 (33.3)	4 (30.8)	6 (28.6)
Arthralgia	1 (16.7)	4 (30.8)	5 (23.8)
Electrocardiogram QT prolonged	1 (16.7)	4 (30.8)	5 (23.8)
Upper respiratory tract infection	1 (16.7)	4 (30.8)	5 (23.8)
Peripheral edema	2 (33.3)	2 (15.4)	5 (23.8)
Anemia	2 (33.3)	1 (7.7)	4 (19.0)
Decreased appetite	3 (50.0)	1 (7.7)	4 (19.0)
Constipation	0 (0.0)	4 (30.8)	4 (19.0)
Pain in extremities	0 (0.0)	4 (30.8)	4 (19.0)

Six patients (28.6%) experienced serious AEs (SAE) including cholangitis, pneumonia, wound infection, confusional state, hydronephrosis, acute respiratory failure, respiratory failure, and a fall. However, no SAEs were considered to be related to treatment. One patient with dedifferentiated CS discontinued treatment because of hydronephrosis. One patient with conventional CS had an AE (a fall) leading to on-treatment death; however, this was deemed to be unrelated to treatment with ivosidenib. Two patients with dedifferentiated CS experienced AEs (acute respiratory failure and respiratory failure) leading to on-treatment death; however, these AEs were deemed to be unrelated to treatment with ivosidenib.

### Clinical activity of ivosidenib in patients with conventional CS

**Figure 2.**
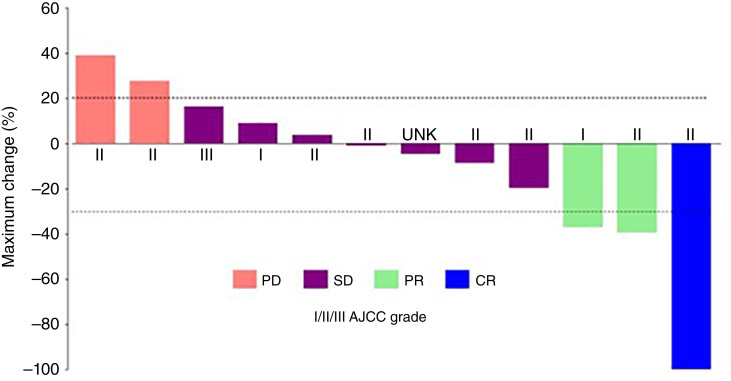
Best percentage change from baseline in target lesion measurement in patients with conventional CS treated with ivosidenib. As per RECIST v1.1, stable disease (SD) occurring with <42 days of the first dose is assigned as unknown (UNK). The American Joint Committee on Cancer (AJCC) grade reported is at screening visit. CR, complete response.

**Figure 3.**
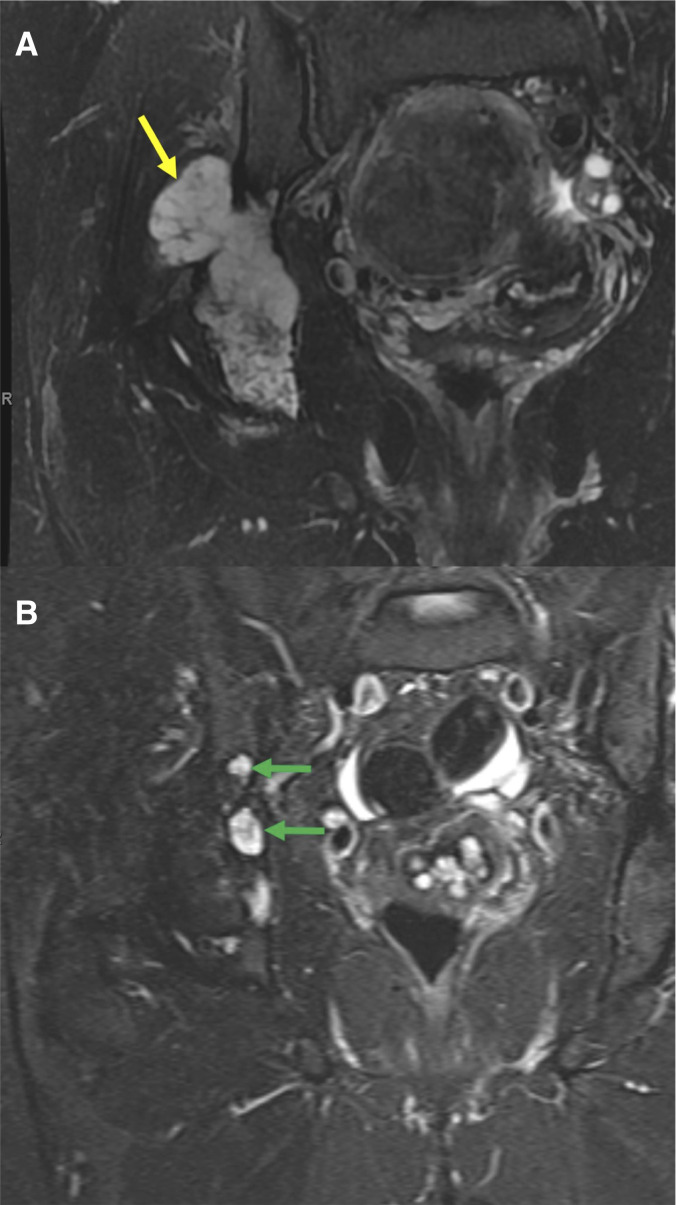
Radiographic images of a patient with conventional CS with PR to ivosidenib at baseline (**A**) and cycle 55 (**B**). Imaging of a 38-year-old female with grade 1 conventional CS (involving the right pelvis) previously treated with one systemic therapy, who then received ivosidenib. Coronal fat–suppressed T2-weighted MRI of the pelvis demonstrated a lobular CS in the right ilium and ischium, with relatively uniform hyperintensity, and a lateral extraosseous component at baseline (yellow arrow). After treatment with ivosidenib, there was reconstitution of the fatty marrow signal in the right ilium, with smaller residual islands of T2-hyperintense foci (green arrows) and regression of the extraosseous component. This resulted in a PR that lasted for 25.8 months.

The ORR according to RECIST (version 1.1; ref. 20) for patients with conventional CS treated with ivosidenib was 23.1% (95% confidence interval: 5.0%–53.8%). The median duration of response was 53.5 months (range: 25.8–67.9 months). One patient, with a lesion at the base of the skull, achieved a complete response with 1,200 mg of ivosidenib once daily (Fig. 2). Two patients achieved PR (one with 500 mg and one with 1,200 mg of ivosidenib once daily; example MRI images of a patient with PR shown in Fig. 3). The tumor response over time of each patient with conventional CS that received ivosidenib is shown in Supplementary Fig. S1. The characteristics of patients with conventional CS achieving response to ivosidenib are described in Supplementary Table S2. Seven patients with conventional CS had stable disease (five received ≥500 mg of ivosidenib once daily), and two had PD (both received ≥500 mg of ivosidenib once daily; Fig. 2). All responses to ivosidenib occurred after >2 years of treatment.

**Figure 4.**
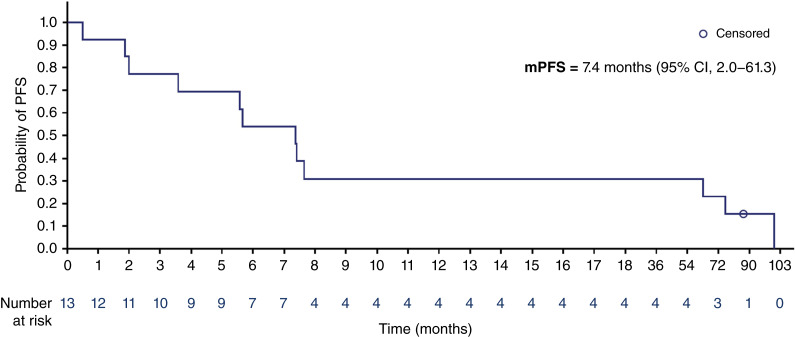
Kaplan–Meier curve for PFS in patients with conventional CS treated with ivosidenib. CI, confidence interval; mPFS, median PFS.

The median PFS of patients with conventional CS treated with ivosidenib was 7.4 months (95% confidence interval, 2.0–61.3 months; Fig. 4). The PFS rates for patients with conventional CS were 53.8% and 30.8% at 6 and 12 months, respectively.

## Discussion

The reported incidence of *IDH1* mutations in CS (12) provides a potential treatment strategy, utilizing m*IDH1* inhibitors such as ivosidenib. In this long-term follow-up study, ivosidenib demonstrated manageable toxicity in patients with m*IDH1* advanced conventional CS, with mostly grade 1 or 2 treatment-emergent AEs. There were no SAEs, treatment discontinuations, dose reductions, or deaths deemed to be associated with ivosidenib treatment. This is consistent with previous studies that have demonstrated the favorable safety profile and tolerability of ivosidenib in solid tumors, such as glioma and cholangiocarcinoma (23, 24).

Earlier analysis of this study demonstrated higher PFS rates in patients without dedifferentiated CS than in patients with dedifferentiated CS; however, survival outcomes by histologic subtype were not a prespecified analysis (20). Therefore, this study investigated the long-term clinical activity of ivosidenib in the subgroup of patients with m*IDH1* advanced conventional CS. Ivosidenib showed promising clinical activity in this subset of patients with CS, with an ORR of 23.1%, a median duration of response of 53.5 months, a 6-month PFS rate of 53.8%, and a median PFS of 7.4 months. The responses to treatment observed in this study were relatively late into treatment with ivosidenib. As chondroid calcifications are frequently observed in CS (25), we hypothesize that this may have provided a delayed observation in response to treatment. Comparatively, in a study comparing first-line systemic therapies in CS, the longest mean PFS was 6.7 months in patients treated with antihormonal therapy.

*IDH1* mutations are an early event in CS carcinogenesis (26); however, this study shows that ivosidenib still demonstrates clinical activity at a later stage. A previous study showed that *IDH1* mutation and subsequent 2-HG production are sufficient to cause enchondromas in mice by inhibiting chondrocyte differentiation (26). Therefore, it is hypothesized that suppression of 2-HG production using ivosidenib may alleviate this differentiation block and consequently provide clinical benefit to patients, even in patients with additional co-mutations. However, it is possible that clinical response to ivosidenib may be affected by the presence of co-mutations. In the present study, baseline co-mutation profiling was only available for one of the three responders, and no co-mutations were detected. So, further study would be required to assess the effect of co-mutations on ivosidenib efficacy.

Other treatment options tested in CS have shown limited success. For example, a previous single-arm, multicenter, phase II study evaluated the activity of pazopanib, a tyrosine kinase inhibitor, in patients with unresectable or metastatic conventional CS (27). Of 47 patients treated with pazopanib, only one patient achieved a PR (27). In this trial, there was a high rate of AEs (98% of patients experienced ≥1 AEs), and 26% had AEs leading to withdrawal from the study (27). Similarly, an investigation into the use of dasatinib in CS, a small-molecule inhibitor of Src kinases, showed limited clinical activity. A phase II trial demonstrated a median PFS of 5.5 months in patients with low- or intermediate-grade CS treated with 100 mg of dasatinib twice daily (28). The 2- and 5-year OS rates for patients with CS in this study were 56% and 9%, respectively (28). Moreover, patients with unresectable, conventional CS treated with dasatinib had a mean PFS of 2.2 months in a study comparing first-line systemic treatments (29). Of the 109 patients receiving dasatinib, 46 and 8 patients had grade 3 and 4 AEs, respectively (29). INBRX-109, a third-generation death receptor 5 agonist, has shown encouraging antitumor activity in patients with unresectable/metastatic CS in a phase I trial (30). However, in this trial, 46.3% of patients had treatment-related AEs, 12.1% of which had an AE of grade ≥3 (30). A phase II, placebo-controlled, randomized trial (NCT04950075) is currently underway to assess the use of INBRX-109 in the management of unresectable or metastatic conventional CS (31). In the absence of standard-of-care treatments, comparative clinical trials are scarce in this disease setting, and only indirect comparisons can be made, but the data reported in this study suggest that clinical activity with ivosidenib is promising.

A limitation of this study was that genomic analysis was incomplete. Future studies with broader exploratory genomic sequencing efforts are warranted to determine if co-mutations affect the clinical activity of ivosidenib for m*IDH1* advanced conventional CS (i.e., it is not known if additional cumulative mutations in cancer drivers or tumor suppressors may overcome any IDH1-directed antitumor activity). Another possible limitation of this study is the less accurate time-dependent assessments of CS grading. In fact, if a screening tumor biopsy could not be obtained, archived primary tumor biopsies, surgical specimens, or biopsies of recurrence or metastases were used. Furthermore, histopathologic diagnoses consistent with CS and radiographic assessments were performed locally at medical centers specialized in sarcomas but were not confirmed centrally. Despite the encouraging results seen in the long-term analysis, the number of patients with m*IDH1* advanced conventional CS was small, and therefore, a relationship between ivosidenib dose and clinical activity could not be established. Additional analysis of clinical trial data with a larger patient population is required to provide further evidence of the tolerability and efficacy of ivosidenib in m*IDH1* advanced conventional CS and determine if there is a relationship between ivosidenib dose and clinical activity. Importantly, OS was not captured in this clinical trial, so a potential survival benefit of ivosidenib cannot be determined from this analysis.

There remains an opportunity for clinical trials to investigate the effect of ivosidenib on survival outcomes in patients with m*IDH1* advanced CS. The ongoing, phase III, international, multicenter, double-blind, randomized, stratified by disease grade (1 vs. 2 vs. 3) and locally advanced versus metastatic disease, placebo-controlled CHONQUER trial (NCT06127407) will address these gaps by investigating the effect of ivosidenib on PFS, OS, and other survival outcomes in patients with m*IDH1* conventional CS not eligible for curative resection (32).

### Conclusions

In conclusion, ivosidenib demonstrated durable disease control and manageable toxicity in patients with m*IDH1* advanced conventional CS, with mostly low-grade treatment-emergent AEs. Ivosidenib has the potential to improve outcomes for patients with m*IDH1* advanced conventional CS who have no available treatment options and is under further evaluation in the phase III CHONQUER trial (NCT06127407).

## Supplementary Material

Supplementary Figure S1 Change in target lesion size from baseline of individual patients with conventional CS receiving ivosidenib.

Supplementary Table S1 Representativeness of study participants.

Supplementary Table S2 Characteristics of patients with conventional CS achieving a response with ivosidenib (N=3).
