# Supplementary material for: Phase I Study of the Mutant IDH1 Inhibitor Ivosidenib: Long-term Safety and Clinical Activity in Patients with Conventional Chondrosarcoma
Source: Clin Cancer Res. 2025 Mar 18;31(11):2108–14. doi: 10.1158/1078-0432.CCR-24-4128 (PMC12130799; doi:10.1158/1078-0432.CCR-24-4128)
Supplement: Supplementary Figure S1 — Change in target lesion size from baseline of individual patients with conventional CS receiving ivosidenib. [file ccr-24-4128_supplementary_figure_s1_suppfs1.docx]

**Supplementary** **Figure 1**. Change in target lesion size from baseline of individual patients with conventional CS receiving ivosidenib.

Partial response

Stable disease

Progressive disease

Complete response

**Best response**
