# Supplementary material for: Phase I Study of the Mutant IDH1 Inhibitor Ivosidenib: Long-term Safety and Clinical Activity in Patients with Conventional Chondrosarcoma
Source: Clin Cancer Res. 2025 Mar 18;31(11):2108–14. doi: 10.1158/1078-0432.CCR-24-4128 (PMC12130799; doi:10.1158/1078-0432.CCR-24-4128)
Supplement: Supplementary Table S1 — Representativeness of study participants. [file ccr-24-4128_supplementary_table_s1_suppts1.docx]

**Supplementary Table 1.** Representativeness of study participants.

| Cancer type | Chondrosarcoma |
| --- | --- |
| Considerations related to: | |
| Sex | There is a higher incidence of chondrosarcoma in males than females. Although differences in incidence between males and females are dependent on the location of chondrosarcoma. |
| Age | The median age of patients with chondrosarcoma is 51, with over 70% of patients over the age of 40 at the time of diagnosis. |
| Race/Ethnicity | Some studies have shown that chondrosarcoma is more prevalent in white patients than in the black population. However, other studies have shown no difference in prevalence between races. |
| Geography | Chondrosarcoma affects approximately 1 person per 200,000 per year in the USA. Whereas in Europe, the incidence is less than in the USA with approximately 1 person per 1,000,000 per year affected with chondrosarcoma. |
| Other Considerations | Characteristics of patients with chondrosarcoma may differ dependent on subtype and location of disease. Data are limited for different subtypes of chondrosarcoma, however approximately 85% of cases are conventional chondrosarcoma. Therefore, the data reported here are for all chondrosarcoma and are applicable to the patient population of this study. |
| Overall representativeness of this study | The age distribution of patients in this study was similar to the age distribution of patients with chondrosarcoma in the literature, with a median age of 55. In the small cohort of chondrosarcoma patients (n=21) reported in this study, there were more male patients (n=13) than female patients (n=8). Most patients with chondrosarcoma in this study cohort were white (n=15), however the race of four patients was unknown/not reported. |
| Sources  <https://journals.lww.com/clinorthop/abstract/2007/06000/osteosarcoma,_chondrosarcoma,_and_ewing_s_sarcoma_.8.aspx> <https://www.cancer.gov/pediatric-adult-rare-tumor/rare-tumors/rare-bone-tumors/chondrosarcoma> <https://www.annalsofoncology.org/article/S0923-7534(19)39658-9/fulltext> <https://www.mdpi.com/2077-0383/12/7/2506> | |
