# Supplementary material for: Phase I Study of the Mutant IDH1 Inhibitor Ivosidenib: Long-term Safety and Clinical Activity in Patients with Conventional Chondrosarcoma
Source: Clin Cancer Res. 2025 Mar 18;31(11):2108–14. doi: 10.1158/1078-0432.CCR-24-4128 (PMC12130799; doi:10.1158/1078-0432.CCR-24-4128)
Supplement: Supplementary Table S2 — Characteristics of patients with conventional CS achieving a response with ivosidenib (N=3). [file ccr-24-4128_supplementary_table_s2_suppts2.docx]

**Supplementary Table 2.** Characteristics of patients with conventional CS achieving a response with ivosidenib (N=3).

|  | | | | | Ivosidenib treatment period | | | | | |
| --- | --- | --- | --- | --- | --- | --- | --- | --- | --- | --- |
| Patient | Ivosidenib dose received | Best response | Prior systemic therapy | Duration of prior systemic therapy (months) | Sum of longest target lesion at baseline (mm) | Maximum change in target lesion from baseline (%) | Duration of treatment (months) | Time to response (months) | Duration of response (months) | Progression-free survival (months) |
| 1 | 500 mg once daily | PR | - | - | 12 | -39.3 | 84.7 | 29.2 | 53.5 | 82.7 |
| 2 | 1200 mg once daily | PR | Amygdalin | - | 111 | -36.9 | 77.4 | 49.8 | 25.8 | 75.6 |
| 3 | 1200 mg once daily | CR | Investigational antineoplastic drugs | 12.1 | 17 | -100.0 | 102.2 | 35.0 | 67.9 | 102.9 |

CR, complete response. mg, milligrams. PR, partial response.
